# Genetically determined gut microbiota associates with pulmonary arterial hypertension: a Mendelian randomization study

**DOI:** 10.1186/s12890-024-02877-2

**Published:** 2024-05-14

**Authors:** Ye Yuan, Shan Li, Manrong Yan, Yan Yang, Changming Zhong, Yijie Hu

**Affiliations:** 1 Department of Cardiovascular Surgery, Daping Hospital, Army Medical University, No.10 Changjiang Branch Road, Yuzhong District, Chongqing, 400042 China; 2 Department of Hepatobiliary and Pancreatic Tumor Center, Chongqing University Cancer Hospital, 181, Hanyu Road, Shapingba District, Chongqing, 400030 China

**Keywords:** Gut microbiota, Pulmonary arterial hypertension, Mendelian randomization, GWAS

## Abstract

**Background:**

Emerging evidences have demonstrated that gut microbiota composition is associated with pulmonary arterial hypertension (PAH). However, the underlying causality between intestinal dysbiosis and PAH remains unresolved.

**Method:**

An analysis using the two-sample Mendelian randomization (MR) approach was conducted to examine the potential causal relationship between gut microbiota and PAH. To assess exposure data, genetic variants associated with 196 bacterial traits were extracted from the MiBioGen consortium, which included a sample size of 18,340 individuals. As for the outcomes, summary statistics for PAH were obtained from the NHGRI-EBI GWAS Catalog, which conducted a meta-analysis of four independent studies comprising a total of 11,744 samples. Causal effects were estimated employing various methods, including inverse variance weighted (IVW), MR-Egger, weighted median, weight mode and simple mode, with sensitivity analyses also being implemented with Cochran’s Q test, MR-Egger intercept test, MR-PRESSO, leave-one-out analysis, and funnel plots.

**Results:**

Following false discovery rate (FDR) correction, the genetically predicted genus *Eubacterium fissicatena group* (odds ratio (OR) 1.471, 95% confidence interval (CI) 1.178–1.837, *q* = 0.076) exhibited a causal association with PAH. In addition, the genus *LachnospiraceaeUCG004* (OR 1.511, 95% CI 1.048–2.177) and genus *RuminococcaceaeUCG002* (OR 1.407, 95% CI 1.040–1.905) showed a suggestive increased risk of PAH, while genus *Eubacterium eligens group* (OR 0.563, 95% CI 0.344–0.922), genus *Phascolarctobacterium* (OR 0.692, 95% CI 0.487–0.982), genus *Erysipelatoclostridium* (OR 0.757, 95% CI 0.579–0.989) and genus *T*–*yzzerella3* (OR 0.768, 95% CI 0.624–0.945) were found to have nominal protective effect against PAH.

**Conclusion:**

The findings from our MR study have revealed a potential causal relationship between gut microbiota and PAH. Specifically, we have identified four types of gut microbiota that exhibit a protective effect on PAH, as well as three types that have a detrimental impact on PAH, thereby offering valuable insights for future mechanistic and clinical investigations in the field of PAH.

**Supplementary Information:**

The online version contains supplementary material available at 10.1186/s12890-024-02877-2.

## Background

Pulmonary hypertension (PH) is characterized by remodeling of the pulmonary artery, resulting in irreversible right heart failure and progressive symptoms that often lead to fatality. The extracellular matrix remodeling and fibrosis in the pulmonary vessels, which correlate with loss of compliance, have been linked to chronic perivascular inflammation and immune dysregulation [1–3]. Among the five subtypes of PH, group 1 is known as pulmonary arterial hypertension (PAH) [4]. It was 73.5% for patients with PAH to survive 5 years without lung transplants [5]. Patients with other subgroups of PH can receive treatment for their underlying conditions [6–8]. However, PAH lacks alternative therapeutic options and can only be managed through drugs that target the carbon monoxide pathway, the endothelin pathway, and the prostacyclin pathway. Despite efforts, no new therapeutic pathways have been proven effective for the treatment of PAH since 2005 [9, 10]. Moreover, treatments for PAH have proven limited in effectiveness to date, and no cure is available.

Emerging evidences suggest that the migration of gut-derived microbes and microbial products to the lungs plays a pivotal role in the pathogenesis of numerous diseases [11, 12], including PAH [13, 14]. Patients with PAH exhibit a greater prevalence of various bacteria that typically promote inflammation, as well as a decreased prevalence of certain species with anti-inflammatory properties, compared to healthy control subjects [13–15]. This phenomenon was similarly observed in animal experiments, and the α diversity of the gut microbiota in different ways induced a decrease in animal models compared to the normal groups [16]. Additionally, the ratio of Firmicutes to Bacteroides increased [16–18], which served as a sensitive biomarker of gut dysbiosis. The aforementioned findings offer substantiation for the notion that the interplay between the gut microbiota and their metabolites serves as the mediator of the intestinal lung axis.

Nevertheless, despite the distinct alterations observed in the intestinal flora of individuals with PAH and animal models in previous studies, the underlying causal connection between intestinal dysbiosis and PAH remains unresolved. Investigating this causality is of significant clinical importance, as it may contribute to understanding the lung-gut axis in PAH development and facilitate the identification of potential therapeutic targets.

In this context, Mendelian randomization (MR) studies offer an approach to address these limitations by genetically evaluating the genuine causal association between exposure and outcome [19]. This methodology effectively mitigates the influence of unobserved confounding variables, as genetic variants are randomly allocated during conception and thus independent of adaptive lifestyle factors and behaviours. This study employed the genome-wide association study (GWAS) summary statistics obtained from the MiBioGen and NHGRI-EBI GWAS Catalog to conduct a two-sample MR analysis, aiming to assess the causal relationship between gut microbiota and PAH.

## Method

### Study design

**Fig. 1:**
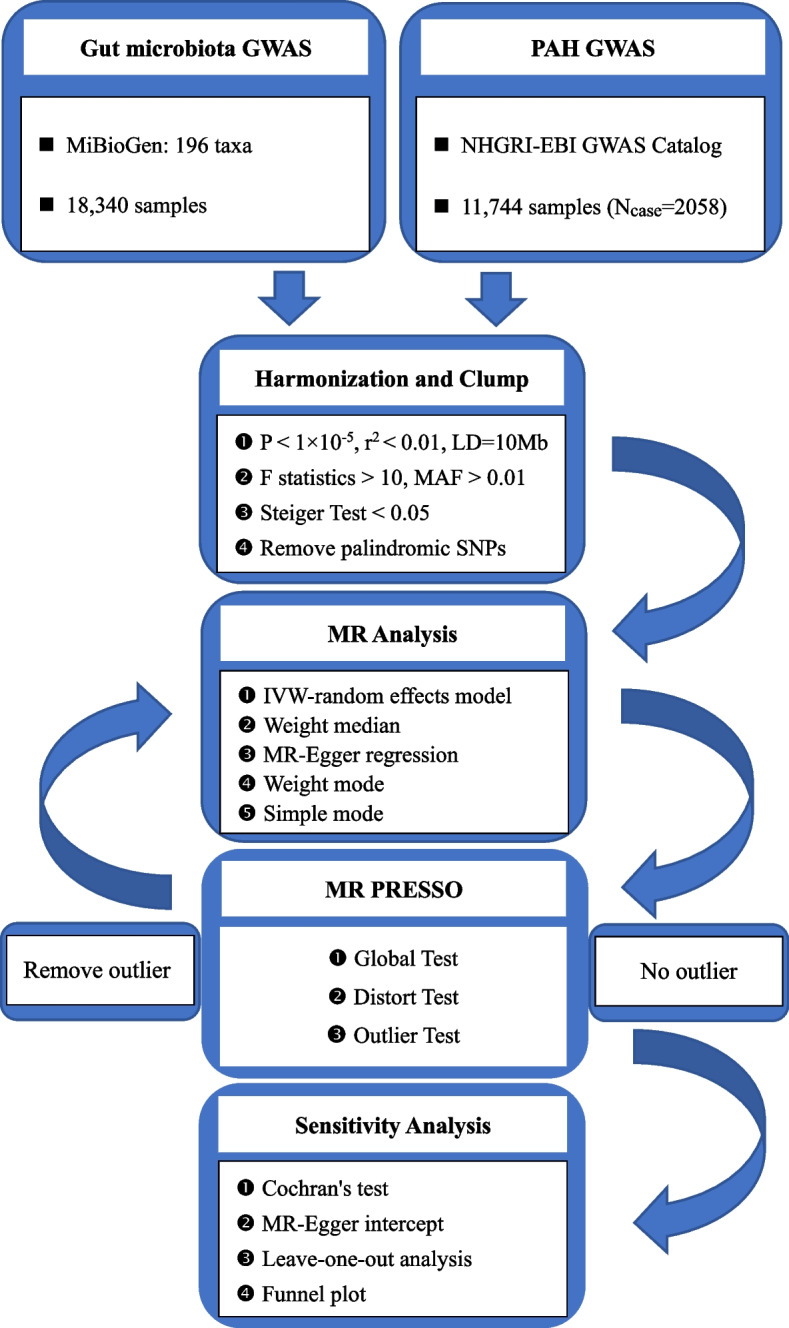
The flow chart of the study. GWAS = genome-wide association study; LD = linkage disequilibrium; MR = Mendelian randomization; MAF = minor allele frequency; MR-PRESSO = Mendelian randomization pleiotropy residual sum and outlier; SNP = single nucleotide polymorphism

A two-sample MR study was performed to assess the causal relationship between the gut microbiota and PAH. An overview of the study description is presented in the figure below (Fig. 1). Simultaneously, adherence to three fundamental assumptions of MR design is crucial to ensure the validity of instrumental variables: 1) instrumental variables (IV), represented by genetic variations, should exhibit a significant correlation with the gut microbiota (exposure); 2) genetic variations must be independent of both known and unknown confounding factors; and 3) there should be no direct correlation between IV and PAH (outcome).

### GWAS data sources

The gut microbiota data utilized in this study were acquired from the international consortium MiBioGen (http://mibiogen.gcc.nl), to our knowledge, which is recognized as the largest publicly accessible sample size Genome-Wide Association Study (GWAS) of the gut microbiome. The datasets encompass the most recent comprehensive meta-analysis of genome-wide proportions, involving 18,340 individuals from 24 population-based cohorts, mostly derived from European populations (*N* = 13,266). Due to the diverse characteristics of age, sex ratio, and diet among cohorts, the researchers employed per-cohort and whole-study filtering methods to determine the taxa included in GWAS analyses.

The summary statistics of PAH were downloaded from the NHGRI-EBI GWAS Catalog (https://www.ebi.ac.uk/gwas) on August 27, 2023 for study GCST007228 [20, 21], which conducted a meta-analysis of four independent studies comprising a total of 11,744 samples (2085 PAH cases). These studies include: 1) the UK National Institute of Health Research Bio-Resource (NIHRBR) for Rare Diseases study, which recruited between January 29, 2003 and January 4, 2017; 2) the US National Biological Sample and Data Repository for Pulmonary Arterial Hypertension/PAH Biobank (PAHB) study, and PAH cases recruited between October 3, 2012 and March 14, 2016; and 3) the Paris Pulmonary Hypertension Allele-Associated Risk cohort (PHAAR) study, all of whom were identified by the French PAH Network from 1 January 2003 to 1 April 2010. Similarly, 4) the British Heart Foundation Pulmonary Arterial Hypertension GWAS (BHFPAH) study was recruited from 3 Dec 1998 to 1 Dec 2011 (Table 1).

**Table 1:** PAH GWAS samples in the study

	Total Cases	PAH Cases	PAH sex ratio (female/male)	Controls	Diagnostic criteria	Populations
NIHRBR	5895	847	579/268	5048	•mPAP > 25 mmHg •PCWP < 15 mmHg •PVR > 3 Woods Units	European
PAHB	2254	694	539/155	1560	•mPAP > 25 mmHg •PCWP < 18 mmHg •PVR > 2.5 Woods Units	European
PHAAR	1337	269	185/84	1068	•mPAP ≥25 mmHg •normal PCWP	European
BHFPAH	2258	275	184/91	1983	•mPAP > 25 mmHg •PCWP < 15 mmHg •PVR > 3 Woods Units	European
Count	11,744	2085	1487/598	9659	―	―

*BHFPAH* British Heart Foundation Pulmonary Arterial Hypertension study: *GWAS* genome-wide association study: *mPAP* mean pulmonary artery pressure: *NIHRBR* National Institute for Health Research BioResource study: *PAH* Pulmonary Arterial Hypertension: *AHB* PAH Biobank study: *PCWP* pulmonary capillary wedge pressure: *PHAAR* Pulmonary Hypertension Allele-Associated Risk study: *PVR* pulmonary vascular resistance

As the present study constitutes a reanalysis of previously published data, the acquisition of supplementary ethical approval was deemed unnecessary.

### SNP selection

To establish the causal association between the gut microbiota and PAH, we employed a rigorous instrumental variable selection process: 1) Given that only a limited number of gut microbiota possessed three or more independent SNPs at the genome-wide significance threshold (*P* < 5 × 10^−8^), we employed a more lenient threshold (*P* < 1 × 10^−5^) to include additional SNPs. This approach was adopted to enhance the availability of SNPs for conducting sensitivity analyses, as in previous studies [22]. 2) The reference panel for calculating the linkage disequilibrium (LD) between SNPs consisted of European sample data from the 1000 Genomes project. Among the SNPs with r^2^ < 0.001 (using a clumping window size of 10 Mb) [23]; 3) SNPs with a minor allele frequency (MAF) of ≤0.01 were excluded from the analyses; 4) To prevent any potential distortion in the orientation of DNA strands or the coding of alleles, palindromic SNPs were eliminated. During the harmonization procedure, the alleles were aligned with the reference sequence of the human genome (build 37), and any SNPs that were ambiguous or duplicated were excluded; 5) The F-statistic was utilized to evaluate the strength of the IVs in relation to exposure characteristics; IVs with F-statistics below 10 were deemed weak and subsequently excluded.

### Statistical analysis

#### MR analysis

The aim of this study was to harmonize the summary statistics of the exposure and outcome datasets to establish a linkage between the effect of the SNP on the exposure and outcome with the same alleles. Based on inverse variance weighted (IVW) for random effects, the primary analysis was conducted, complemented by MR-Egger regression, weighted median, weighted mode and simple mode to ascertain causality [24]. The supplementary analyses were chosen for their ability to yield more reliable estimates across a broader spectrum of scenarios. The MR-Egger regression method relies on the assumption of instrument strength independent of direct effect, enabling the assessment of pleiotropy through the intercept. A zero intercept suggests the absence of horizontal pleiotropy, indicating consistency between MR-Egger regression and IVW [25]. In contrast, the weighted median method permits accurate estimation of causal associations even when up to 50% of instrumental variables are invalid, and if the InSIDE hypothesis is violated, the weighted-model estimation method exhibits greater detection ability with regard to causal effect, less deviation, and a lower type I error rate than MR-Egger regression [26].

Furthermore, a correction for the false discovery rate (FDR) was implemented using the Benjamin Hochberg procedure, employing a stringent FDR threshold of *q* < 0.1.

#### Sensitivity analysis

Multiple sensitivity analyses were employed to validate the findings. First, Cochran’s Q test for heterogeneity was utilized. Additionally, MR-Pleiotropy Residual Sum and Outlier (MR-PRESSO) analysis was conducted to assess horizontal pleiotropy and exclude SNPs with outliers, thereby reducing the impact of pleiotropy on causal effects [27]. In our study, if significant horizontal pleiotropy was detected in the MR-PRESSO test, SNPs identified as outliers (*P* < 0.05) were removed, and the remaining SNPs were re-evaluated in the IVW analysis. Second, the MR-Egger regression intercept was employed to estimate the potential presence of pleiotropy in SNPs, where a *P* value > 0.05 suggests the absence of horizontal pleiotropy. Third, further strengthen the robustness of the results with leave-one-out analysis. In addition, we tested whether the causal direction inferred was correct by applying the MR Steiger test for directionality [28].

All statistical analyses were conducted utilizing the TwoSampleMR (version 0.5.7), MR-PRESSO (version 1.0), psych (version 2.3.6) and ggplot2 (version 3.4.3) packages in R version 4.3.0, developed by the R Foundation for Statistical Computing in Vienna, Austria.

## Results

In the present study, a total of 196 gut microbiota taxa were identified, encompassing five biological levels (119 genera, 32 families, 20 orders, 16 classes, and 9 phyla). Supplementary material provided detailed information on the final SNPs for each bacterial trait, including effect allele, other allele, beta, standard error, and *P* value. The F values of the selected SNPs ranged from 14.59 to 88.43, suggesting the absence of any weak instrument bias (Additional file 1: Table S1).

**Fig. 2:**
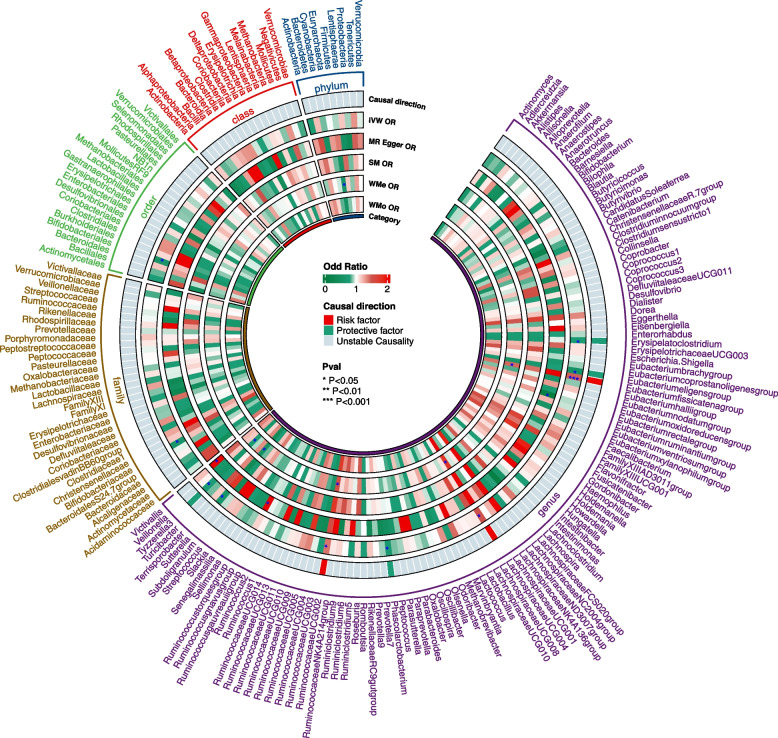
Main MR result of causal association of gut microbiota on PAH. A circular heatmap visually displays the 196 taxa that exhibit significant differences between gut microbiota with PAH. Each segment of the heatmap corresponds to a distinct taxon, with its name labeled along the outer circumference of the chart. The color scheme is provided in the center of the panel, while the explanatory information for each layer is depicted within the gaps of the fig. SM = simple mode; WMe = weighted median; WMo = weighted mode

We identified a total of 108, 188, 229, 375, and 1287 SNPs at the phylum, class, order, family, and genus levels, respectively, from the filtered set of independent variables (Additional file 1: Table S1). Overall, the IVW estimation indicated a genetic prediction of 10 bacterial taxa showing an association with PAH (Additional file 1: Table S2, S3). However, the order *Bifidobacteria*, family *Bifidobacteriaceae*, and genus *Sutterella* were identified as being linked to PAH through MR-Egger analysis yielded contradictory results, implying that this causal relationship lacks validity [23]. Consequently, seven taxa were substantiated to possess causal effects with PAH (Fig. 2, Additional file 1: Table S3).

**Fig. 3:**
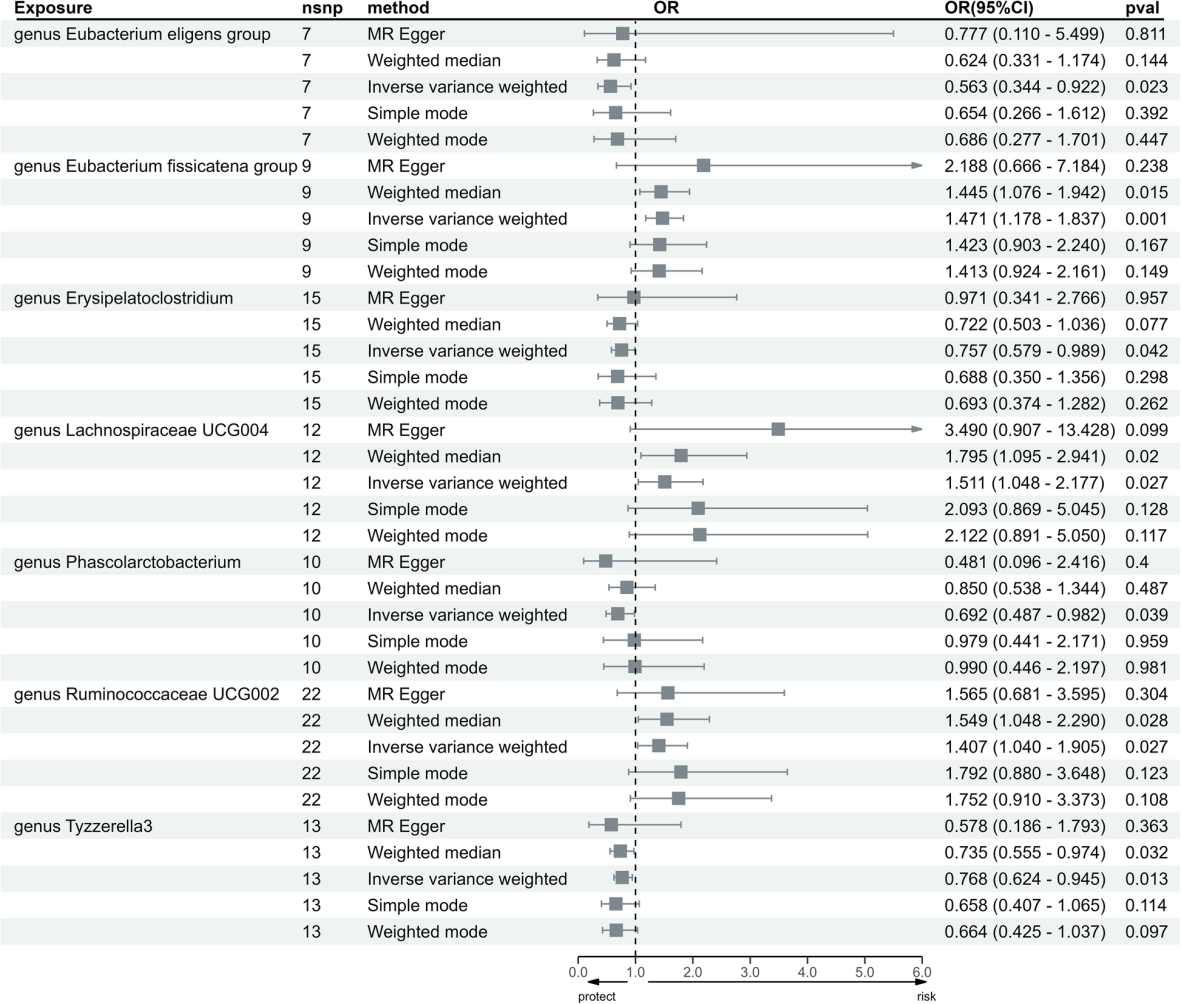
Causal effects of the gut microbiota on PAH

Following the application of FDR correction, the significant result was that the genus *Eubacterium fissicatena group* (OR 1.471, 95% CI 1.178–1.837, *P* = 6.602 × 10^−4^, *q* = 0.076) exhibited a positive association with PAH. Additionally, MR analyses revealed the inclusion of two bacterial features suggestive of an increased risk of PAH, including genus *LachnospiraceaeUCG004* (OR 1.511, 95% CI 1.048–2.177, *P* = 0.027, *q* = 0.520) and genus *RuminococcaceaeUCG002* (OR 1.407, 95% CI 1.040–1.905, *P* = 0.038, *q* = 0.521). Four additional bacterial groups were found to have nominal protective effects against PAH: genus *Eubacterium eligens group* (OR 0.563, 95% CI 0.344–0.922, *P* = 0.023, q = 0.506); genus *Phascolarctobacterium* (OR 0.692, 95% CI 0.487–0.982, *P* = 0.039, *q* = 0.592); genus *Erysipelatoclostridium* (OR 0.757, 95% CI 0.579–0.989, *P* = 0.042, *q* = 0.602); and genus *Tyzzerella3* (OR 0.768, 95% CI 0.624–0.945, *P* = 0.013, *q* = 0.446) (Fig. 3, Additional file 2: Fig. S3).

After removing the identified outliers (rs76973485, rs12336782, rs17785622, rs12996055), we reperformed MR analysis. Through Cochran’s Q and MR-PRESSO, no significant heterogeneity (*P* > 0.05) or outliers were detected. Furthermore, all *P* values of MR-Egger interpretation were > 0.05, showing the absence of horizontal pleiotropy. Additionally, no instruments were removed based on Steiger filtering.

(*P*_steiger_ < 0.05), and leave-one-out analysis also revealed the robustness of our main results. Finally, we performed MR visualization methods, including forest plots, leave-one-out analysis, funnel plots, and scatter plots, to evaluate the robustness of the results (Fig. 3, Additional file 2: Figs. S1–S3).

## Discussion

The present study employs GWAS datasets to conduct a two-sample MR analysis on summary statistics to genetically determine the causal relationship between the gut microbiota and PAH. Our findings indicate that the bacterial genus *Eubacterium fissicatena group* exhibits a causal relationship with an increased risk of PAH, whereas the genera *LachnospiraceaeUCG004* and *RuminococcaceaeUCG002* demonstrate a nominal causal association with PAH risk. Furthermore, we identify four additional bacterial groups, namely genus *Eubacterium eligens group*, genus *Phascolarctobacterium*, genus *Erysipelatoclostridium*, and genus *Tyzzerella3*, which exhibit nominal protective effects against PAH.

Inflammation, an important pathogenic factor of PAH that exhibits a causal correlation with the extent of pulmonary vascular remodeling, is widely accepted [29–31]. The gut-lung axis has emerged to underscore the interplay between gut microbes and inflammation in the lung. The causality of these effects in promoting PAH remains uncertain, as they could potentially arise as a result of gut hypoxia or hypoperfusion. Nevertheless, recent evidences indicated that alterations in the gut microbiota were more inclined to play a role in the pathogenesis of pulmonary hypertension, given that variations in the gut microbiota composition have been observed across various disease stages [13–18]. Notably, Moutsoglou and colleagues conducted a study comprising a cohort of 73 patients with PAH, 15 family control subjects, and 39 healthy individuals [13]. The findings of their study revealed a distinct gut microbial profile in PAH patients, characterized by decreased levels of anti-inflammatory short-chain fatty acids (SCFAs) and secondary bile acids in their plasma. Additionally, the study demonstrated that there existed no discernible correlation between the Shannon diversity index and serum N-terminal pro–brain natriuretic peptide, RV global longitudinal strain, or RV free wall strain.

SCFAs are the main metabolites produced by specific intestinal flora following the fermentation of dietary fiber and resistant starch, representing a global anti-inflammatory impact through the upregulation of anti-inflammatory cytokines and the downregulation of proinflammatory cytokines via various mechanisms, thereby facilitating the maintenance of mucosal homeostasis [32–35]. They interact with G-protein-coupled receptors (GPRs), specifically GPR43, GPR41, and GPR109a, to modulate a variety of host responses, including inflammation, intestinal barrier integrity, and energy homeostasis. The primary mechanism by which SCFAs suppress inflammation is through the inhibition of the NF-κB pathway and/or histone deacetylase (HDAC) function, resulting in the downregulation of proinflammatory cytokines such as TNF-α, IL-6, IL-12, and IFN-γ and the upregulation of anti-inflammatory cytokines such as IL-10 and TGF-β [36–40].

The genus *Eubacterium,* as a major butyrate producer, exhibits the potential to induce anti-inflammatory effects. *Eubacterium eligens* is an important Eubacterium found in the human colon that promotes the production of the anti-inflammatory cytokine IL-10 [41, 42]. In a recent study, a lower abundance of *Eubacterium eligens* and reductions in circulating SCFAs were observed in PAH patients [13, 14]. Therefore, *Eubacterium eligens* may produce SCFAs to exert their anti-proinflammatory effects, thereby preventing PAH initiation and development [43]. In the present study, the genus *Eubacterium eligens* was also observed to exhibit a protective effect against PAH, whereas the genus *Eubacterium fissicatena* group seemed to increase the risk of developing PAH. The *Eubacterium fissicatena* is categorized as an opportunistic pathogen that negatively correlates with propionate and butyrate [44], thereby inducing inflammation. Intriguingly, comparable findings were also observed in hypertensive patients, with normotensive individuals displaying elevated levels of *Eubacterium eligens,* while essential hypertensive subjects exhibited high levels of *Eubacterium fissicatena* [45]. Taken together, some gut microbiota may exert SCFA-related anti-proinflammatory effects by preventing PAH initiation and development.

However, it is worth noting that not all SCFA microbial producers possess beneficial features. The families *Lachnospiraceae* and *Ruminococcaceae* possess the capacity to produce butyrate and other SCFAs through distinct biosynthetic pathways [46, 47]. In contrast, the findings from our MR analysis revealed that both the genus *Lachnospiraceae UCG004* and the genus *Ruminococcaceae UCG002* were suggestively associated with increased risks of inducing PAH. The abundance of the two taxa also exhibits an increase within the intestinal lumen of individuals afflicted with various diseases and elderly individuals [48–52]. However, members of this family have consistently demonstrated their capacity to generate favorable metabolites to the host.

However, this study is subject to certain limitations, which necessitate a more cautious interpretation of the findings. The first limitation is the inability to conduct subgroup analysis, such as assessing the severity of pulmonary arterial hypertension (PAH), due to the unavailability of individual level data. This is significant as alterations in intestinal flora among PAH patients were not found to be associated with changes in right heart function [13]. In addition, the limitation of the exposure dataset to the genus level hinders our ability to investigate the causal relationship between gut microbiota and PAH at the species level. Furthermore, the predominance of participants of European descent in genome-wide association studies restricts the generalizability of our findings to other populations. Finally, while our findings establish a causal association between specific gut microbiota and PAH, further research is needed to elucidate the underlying mechanisms.

## Conclusions

The results of our MR study identified a potential causal effect of the gut microbiota and PAH. This valuable finding probably contributes to the identification of specific intestinal bacteria as biomarkers for pulmonary PAH, as well as clinical prevention and intervention of PAH through the implementation of fecal microbiota transplantation. Together, despite the presence of compelling evidence connecting gut dysbiosis to the initial development of PAH, the utilization of intestinal microbiota as a therapeutic intervention in clinical settings still requires significant advancements. It is imperative to conduct meticulous experimental investigations to establish the causative relationship between gut dysbiosis, altered gut microbiome, and the pathogenesis of PAH prior to considering the modulation of gut microbiota as a viable therapeutic approach for treating PAH.

### Supplementary Information

**Additional file 1.**

**Additional file 2.**

**Additional file 3.**

## Data Availability

All data generated or analyzed during this study are included in this published article and its supplementary information files.

## Abbreviations

BHFPAH: British Heart Foundation Pulmonary Arterial Hypertension study
CI: Confidence interval
FDR: False discovery rate
GPRs: G-protein-coupled receptors
GWAS: Genome-wide association studies
HDAC: Histone deacetylase
IV: Instrumental variables
IVW: Inverse variance weighted
LD: Linkage disequilibrium
mPAP: Mean pulmonary artery pressure
MAF: Minor allele frequency
MR: Mendelian randomization
MR-PRESSO: MR pleiotropy residual sum and outlier
NIHRBR: National Institute for Health Research BioResource study
OR: Odds ratio
PAH: Pulmonary arterial hypertension
PAHB: PAH Biobank study
PCWP: pulmonary capillary wedge pressure
PH: Pulmonary hypertension
PHAAR: Pulmonary Hypertension Allele-Associated Risk study
PVR: Pulmonary vascular resistance
SCFA: Short-chain fatty acid
SNP: Single nucleotide polymorphisms
