# Supplementary material for: Genetically determined gut microbiota associates with pulmonary arterial hypertension: a Mendelian randomization study
Source: BMC Pulm Med. 2024 May 14;24:235. doi: 10.1186/s12890-024-02877-2 (PMC11094871; doi:10.1186/s12890-024-02877-2)
Supplement: Supplementary file 2 — Additional file 2. [file 12890_2024_2877_MOESM2_ESM.docx]

**Genetically Determined Gut Microbiota Associated with Pulmonary Arterial Hypertension: A Mendelian Randomization Study**

Ye Yuan^1^, Shan Li^2^, Manrong Yan^1^, Yan Yang^1^, Changming Zhong^1^, Yijie Hu ^1*^

1. Department of Cardiovascular Surgery, Daping Hospital, Army Medical University, No.10 Changjiang Branch Road, Yuzhong District, Chongqing, 400042, China.

2. Department of Hepatobiliary and Pancreatic Tumor Center, Chongqing University Cancer Hospital, 181, Hanyu Road, Shapingba District, Chongqing 400030, China.

***Correspondence:**

Yijie Hu, MD, Ph.D, Department of Cardiovascular Surgery, Daping Hospital, Army Medical University, No.10 Changjiang Branch Road, Yuzhong District, Chongqing, 400042, China. Tel: +86-23-68729521; E-mail: yijie.hu@tmmu.edu.cn

**Additional file 2**

Figure S1. Funnel plots for MR analyses of the causal effect of gut microbiota on pulmonary arterial hypertension.

Figure S2. Leave-one-out sensitivity based on IVW model for gut microbiota on pulmonary arterial hypertension.

Figure S3. Scatter plots for MR analyses of the causal effect of gut microbiota on pulmonary arterial hypertension.


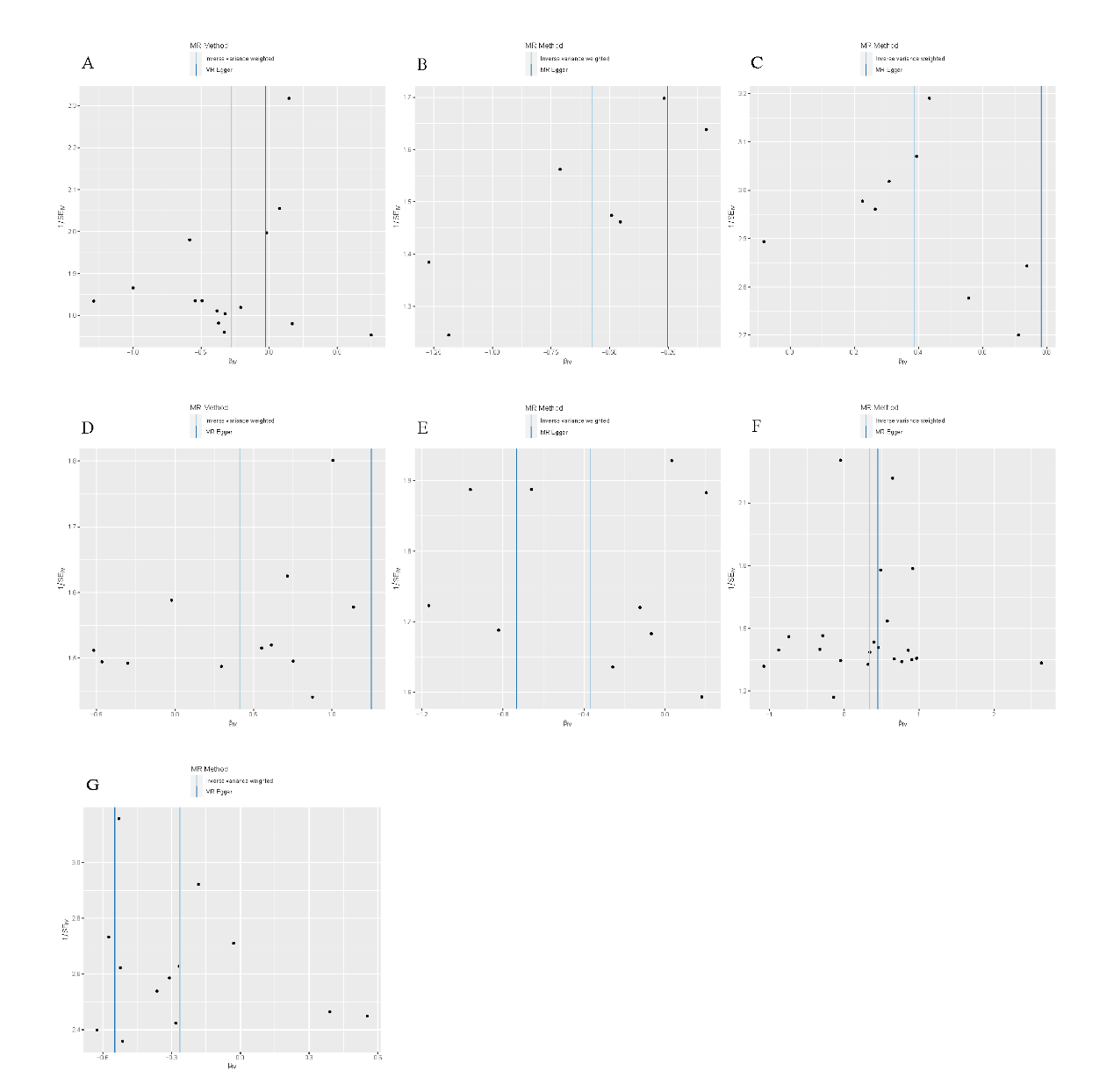


Figure S1. Funnel plots for MR analyses of the causal effect of gut microbiota on pulmonary arterial hypertension.

A: genus *Phascolarctobacterium*; B: genus *LachnospiraceaeUCG004*; C: genus *Eubacterium fissicatenag roup*; D: genus *Eubacterium eligens group*; E: genus *Erysipelatoclostridium*; F: genus *Tyzzerella3*; G: genus *RuminococcaceaeUCG002*

*
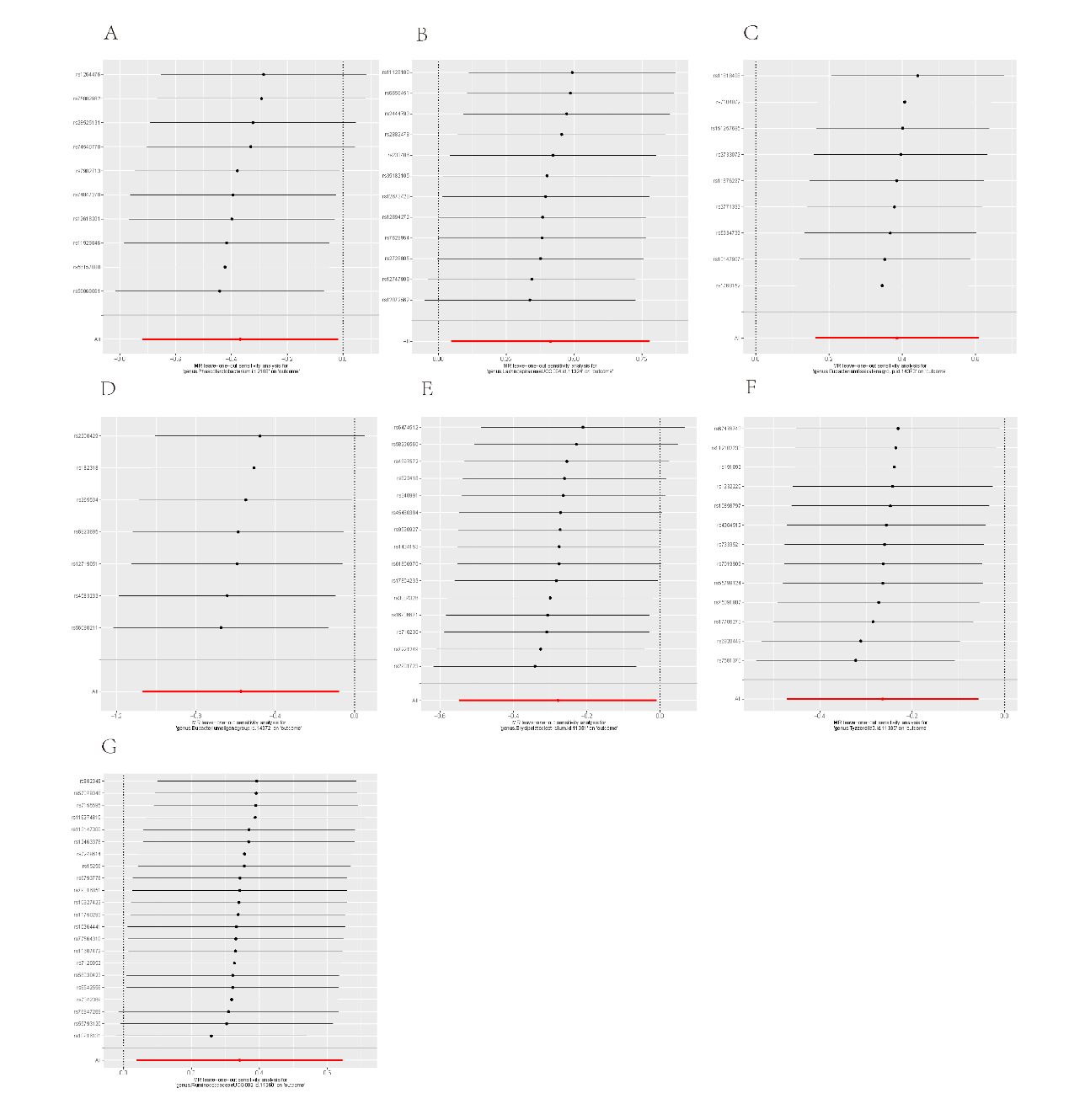
*

Figure S2. Leave-one-out sensitivity based on IVW model for gut microbiota on pulmonary arterial hypertension.

A: genus *Phascolarctobacterium*; B: genus *LachnospiraceaeUCG004*; C: genus *Eubacterium fissicatenag roup*; D: genus *Eubacterium eligens group*; E: genus *Erysipelatoclostridium*; F: genus *Tyzzerella3*; G: genus *RuminococcaceaeUCG002*


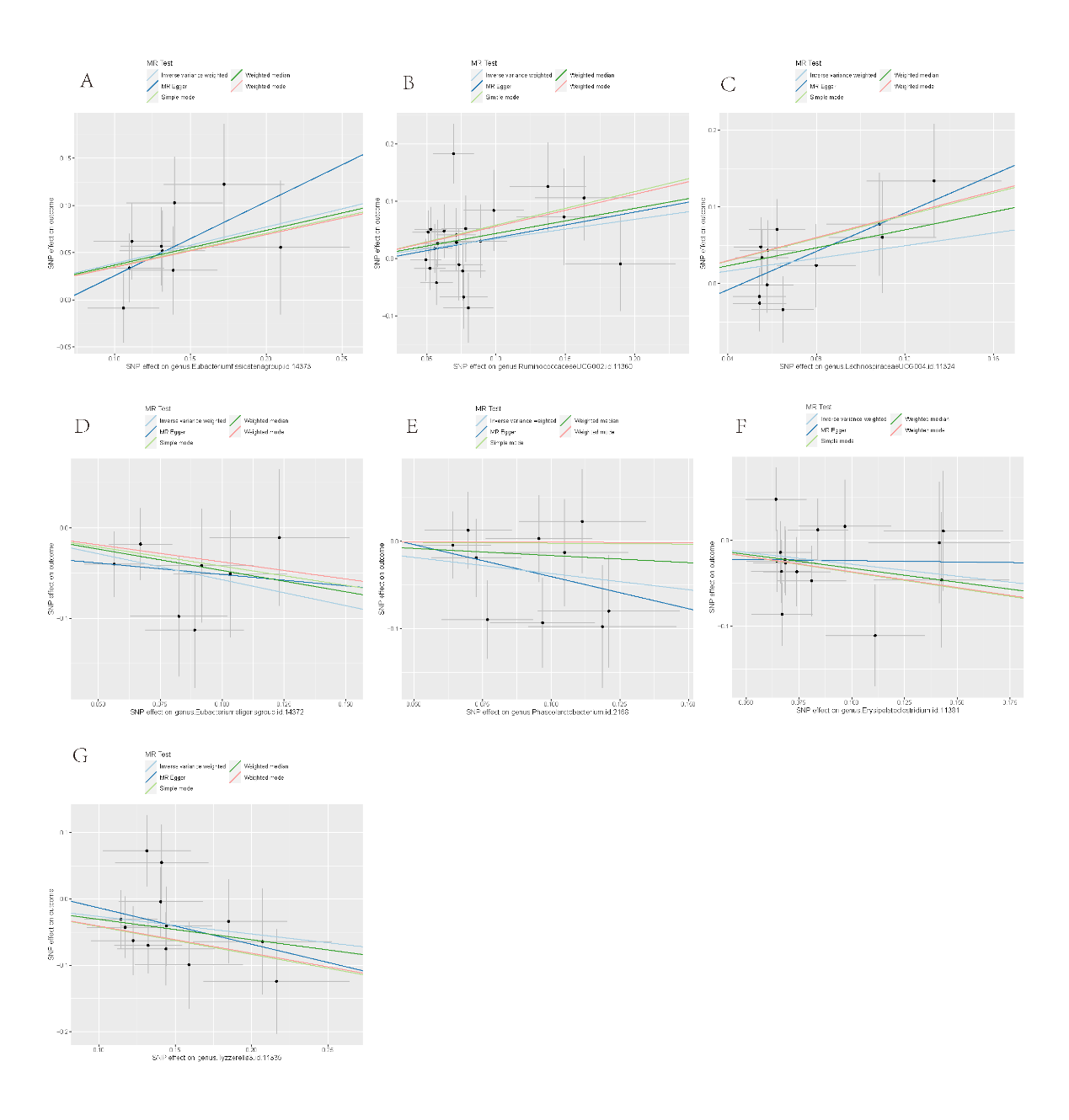


Figure S3. Scatter plots for MR analyses of the causal effect of gut microbiota on pulmonary arterial hypertension.

A: genus *Eubacterium fissicatenag roup*; B: genus *RuminococcaceaeUCG002*; C: genus *LachnospiraceaeUCG004*; D: genus *Eubacterium eligens group*; E: genus *Phascolarctobacterium*; F: genus *Erysipelatoclostridium*; G: genus *Tyzzerella3*
