# Supplementary material for: Genetically determined gut microbiota associates with pulmonary arterial hypertension: a Mendelian randomization study
Source: BMC Pulm Med. 2024 May 14;24:235. doi: 10.1186/s12890-024-02877-2 (PMC11094871; doi:10.1186/s12890-024-02877-2)
Supplement: Supplementary file 3 — Additional file 3. [file 12890_2024_2877_MOESM3_ESM.docx]

**STROBE-MR checklist of recommended items to address in reports of Mendelian randomization studies**^1^ ^2^

| **Item No.** | **Section** | **Checklist item** | **Page No.** | **Relevant text from manuscript** |
| --- | --- | --- | --- | --- |
| 1 | **TITLE and ABSTRACT** | Indicate Mendelian randomization (MR) as the study’s design in the title and/or the abstract if that is a main purpose of the study | 1  2 | **TITLE**: Genetically determined gut microbiota associates with pulmonary arterial hypertension: a Mendelian randomization study.  **ABSTRACT**: An analysis using the two-sample Mendelian randomization (MR) approach was conducted to examine the potential causal relationship between gut microbiota and PAH. |
|  | **INTRODUCTION** |  |  |  |
| 2 | **Background** | Explain the scientific background and rationale for the reported study. What is the exposure? Is a potential causal relationship between exposure and outcome plausible? Justify why MR is a helpful method to address the study question | 4-5 | ➊ Pulmonary hypertension (PH) is characterized by remodeling of the pulmonary artery, resulting in irreversible right heart failure and progressive symptoms that often lead to fatality. Despite efforts, no new therapeutic pathways have been proven effective for the treatment of PAH since 2005. Moreover, treatments for PAH have proven limited in effectiveness to date, and no cure is available.  ➋ Nevertheless, despite the distinct alterations observed in the intestinal flora of individuals with PAH and animal models in previous studies, the underlying causal connection between intestinal dysbiosis and PAH remains unresolved.  ➌ In this context, Mendelian randomization (MR) studies offer an approach to address these limitations by genetically evaluating the genuine causal association between exposure and outcome. |
| 3 | **Objectives** | State specific objectives clearly, including pre-specified causal hypotheses (if any). State that MR is a method that, under specific assumptions, intends to estimate causal effects | 5 | aiming to assess the causal relationship between gut microbiota and PAH. |
|  | **METHODS** |  |  |  |
| 4 | **Study design and data sources** | Present key elements of the study design early in the article. Consider including a table listing sources of data for all phases of the study. For each data source contributing to the analysis, describe the following: | 7 | ➊ The gut microbiota data utilized in this study were acquired from the international consortium MiBioGen (<http://mibiogen.gcc.nl>)  ➋ The summary statistics of PAH were downloaded from the NHGRI-EBI GWAS Catalog (<https://www.ebi.ac.uk/gwas>) |
|  | a) | Setting: Describe the study design and the underlying population, if possible. Describe the setting, locations, and relevant dates, including periods of recruitment, exposure, follow-up, and data collection, when available. | 6―8 | ➊An overview of the study description is presented in the figure below (Figure.1).  ➋The gut microbiota data utilized in this study were acquired from the international consortium MiBioGen  ➌ The summary statistics of PAH were downloaded from the NHGRI-EBI GWAS Catalog (<https://www.ebi.ac.uk/gwas>). In addition, lines 100 to 110 contain detailed information about the study population regarding recruitment, diagnosis, and population structure. |
|  | b) | Participants: Give the eligibility criteria, and the sources and methods of selection of participants. Report the sample size, and whether any power or sample size calculations were carried out prior to the main analysis | 7―8 | ➊ The gut microbiota data utilized in this study were acquired from the international consortium MiBioGen (<http://mibiogen.gcc.nl>), involving 18,340 individuals from 24 population-based cohorts. Due to the diverse characteristics of age, sex ratio, and diet among cohorts, the researchers employed per-cohort and whole-study filtering methods to determine the taxa included in GWAS analyses.  ➋The summary statistics of PAH were downloaded from the NHGRI-EBI GWAS Catalog (<https://www.ebi.ac.uk/gwas>) on August 27, 2023 for study GCST007228, which conducted a meta-analysis of four independent studies comprising a total of 11,744 samples (2,085 PAH cases).  ➌ The recruitment method, eligibility criteria and population of the study subjects can be found in Table 1 and in the supplementary material of the literature (20th and 21th) |
|  | c) | Describe measurement, quality control and selection of genetic variants | 8―9 | Please refer to the SNP selection in the methods section of manuscript, as well as to document in Additional file1 of our supplementary material uploaded. |
|  | d) | For each exposure, outcome, and other relevant variables, describe methods of assessment and diagnostic criteria for diseases | 5, 8 | ➊ MR design is crucial to ensure the validity of instrumental variables: 1) instrumental variables (IV), represented by genetic variations, should exhibit a significant correlation with the gut microbiota (exposure); 2) genetic variations must be independent of both known and unknown confounding factors; and 3) there should be no direct correlation between IV and PAH (outcome).  ➋Please refer to the Table 1and literature 20th |
|  | e) | Provide details of ethics committee approval and participant informed consent, if relevant | 7  8 | ➊ The datasets encompass the most recent comprehensive meta-analysis of genome-wide proportions, involving 18,340 individuals from 24 population-based cohorts, mostly derived from European populations (N=13266).  ➋ Table 1 shows that all PAH GWAS populations are of European ancestry. |
| 5 | **Assumptions** | Explicitly state the three core IV assumptions for the main analysis (relevance, independence and exclusion restriction) as well assumptions for any additional or sensitivity analysis | 5  9  10 | ➊Simultaneously, adherence to three fundamental assumptions of MR design is crucial to ensure the validity of instrumental variables.  ➋ Based on inverse variance weighted (IVW) for random effects, the primary analysis was conducted, complemented by MR-Egger regression, weighted median, weighted mode and simple mode to ascertain causality.  ➌ Furthermore, a correction for the false discovery rate (FDR) was implemented using the Benjamin Hochberg procedure, employing a stringent FDR threshold of q < 0.1.  ➍ Multiple sensitivity analyses were employed to validate the findings. |
| 6 | **Statistical methods: main analysis** | Describe statistical methods and statistics used |  |  |
|  | a) | Describe how quantitative variables were handled in the analyses (i.e., scale, units, model) | 9 | Based on inverse variance weighted (IVW) for random effects, the primary analysis was conducted. |
|  | b) | Describe how genetic variants were handled in the analyses and, if applicable, how their weights were selected | 8 | Please refer to the paragraph under the sub-heading "SNP SLECTION" and Figure 1. |
|  | c) | Describe the MR estimator (e.g. two-stage least squares, Wald ratio) and related statistics. Detail the included covariates and, in case of two-sample MR, whether the same covariate set was used for adjustment in the two samples | 9, 10 | ➊ The aim of this study was to harmonize the summary statistics of the exposure and outcome datasets to establish a linkage between the effect of the SNP on the exposure and outcome with the same alleles.  ➋ Based on inverse variance weighted (IVW) for random effects, the primary analysis was conducted, complemented by MR-Egger regression, weighted median, weighted mode and simple mode to ascertain causality. |
|  | d) | Explain how missing data were addressed | 8 | As the present study constitutes a reanalysis of previously published data, the acquisition of supplementary ethical approval was deemed unnecessary. |
|  | e) | If applicable, indicate how multiple testing was addressed |  | NA |
| 7 | **Assessment of assumptions** | Describe any methods or prior knowledge used to assess the assumptions or justify their validity | 8  9  10  11 | ➊ The F-statistic was utilized to evaluate the strength of the IVs in relation to exposure characteristics; IVs with F-statistics below 10 were deemed weak and subsequently excluded.  ➋ Furthermore, a correction for the false discovery rate (FDR) was implemented using the Benjamin Hochberg procedure, employing a stringent FDR threshold of q < 0.1.  ➌In addition, we tested whether the causal direction inferred was correct by applying the MR Steiger test for directionality.  ➍ However, the order Bifidobacteria, family Bifidobacteriaceae, and genus Sutterella were identified as being linked to PAH through MR-Egger analysis yielded contradictory results, implying that this causal relationship lacks validity. |
| 8 | **Sensitivity analyses and additional analyses** | Describe any sensitivity analyses or additional analyses performed (e.g. comparison of effect estimates from different approaches, independent replication, bias analytic techniques, validation of instruments, simulations) | 10 | ➊First, Cochran's Q test for heterogeneity was utilized. Additionally, MR-Pleiotropy Residual Sum and Outlier (MR-PRESSO) analysis was conducted to assess horizontal pleiotropy and exclude SNPs with outliers, thereby reducing the impact of pleiotropy on causal effects. if significant horizontal pleiotropy was detected in the MR-PRESSO test, SNPs identified as outliers (P < 0.05) were removed, and the remaining SNPs were re-evaluated in the IVW analysis.  ➋Second, the MR-Egger regression intercept was employed to estimate the potential presence of pleiotropy in SNPs, where a P value > 0.05 suggests the absence of horizontal pleiotropy.  ➌Third, further strengthen the robustness of the results with leave-one-out analysis. In addition, we tested whether the causal direction inferred was correct by applying the MR Steiger test for directionality. |
| 9 | **Software and pre-registration** |  |  |  |
|  | a) | Name statistical software and package(s), including version and settings used | 11 | The analyses were conducted utilizing the TwoSampleMR (version 0.5.7), MR-PRESSO (version 1.0), psych (version 2.3.6) and ggplot2 (version 3.4.3) packages in R version 4.3.0, developed by the R Foundation for Statistical Computing in Vienna, Austria. |
|  | b) | State whether the study protocol and details were pre-registered (as well as when and where) |  | not registered |
|  | **RESULTS** |  |  |  |
| 10 | **Descriptive data** |  |  |  |
|  | a) | Report the numbers of individuals at each stage of included studies and reasons for exclusion. Consider use of a flow diagram | 6 | Figure 1. The flow chart of the study |
|  | b) | Report summary statistics for phenotypic exposure(s), outcome(s), and other relevant variables (e.g. means, SDs, proportions) |  | Additional file 1: Table S1. |
|  | c) | If the data sources include meta-analyses of previous studies, provide the assessments of heterogeneity across these studies |  | NA |
|  | d) | For two-sample MR:  i.  Provide justification of the similarity of the genetic variant-exposure associations between the exposure and outcome samples  ii.  Provide information on the number of individuals who overlap between the exposure and outcome studies |  | Additional file 1: Table S1.  Through the examination of the recruitment sites and populations presented in the gut microbiota GWAS and PAH GWAS study cohorts, it is evident that there is no substantial overlap of participants between the exposure data and the outcome dataset. |
| 11 | **Main results** |  |  |  |
|  | a) | Report the associations between genetic variant and exposure, and between genetic variant and outcome, preferably on an interpretable scale |  | The F values of the selected SNPs ranged from 14.59 to 88.43, suggesting the absence of any weak instrument bias (Additional file 1: Table S1). |
|  | b) | Report MR estimates of the relationship between exposure and outcome, and the measures of uncertainty from the MR analysis, on an interpretable scale, such as odds ratio or relative risk per SD difference | 12  12  13 | ➊ Following the application of FDR correction, the significant result was that the genus Eubacterium fissicatena group (OR 1.471, 95% CI 1.178–1.837, P=6.602×10-4, q=0.076) exhibited a positive association with PAH.  ➋Additionally, MR analyses revealed the inclusion of two bacterial features suggestive of an increased risk of PAH, including genus LachnospiraceaeUCG004 (OR 1.511, 95% CI 1.048–2.177, P=0.027, q=0.520) and genus RuminococcaceaeUCG002 (OR 1.407, 95% CI 1.040–1.905, P=0.038, q=0.521).  ➌ Four additional bacterial groups were found to have nominal protective effects against PAH: genus Eubacterium eligens group (OR 0.563, 95% CI 0.344–0.922, P=0.023, q=0.506); genus Phascolarctobacterium (OR 0.692, 95% CI 0.487–0.982, P=0.039, q=0.592); genus Erysipelatoclostridium (OR 0.757, 95% CI 0.579–0.989, P=0.042, q=0.602); and genus Tyzzerella3 (OR 0.768, 95% CI 0.624–0.945, P=0.013, q=0.446) |
|  | c) | If relevant, consider translating estimates of relative risk into absolute risk for a meaningful time period |  | NA |
|  | d) | Consider plots to visualize results (e.g. forest plot, scatterplot of associations between genetic variants and outcome versus between genetic variants and exposure) | 12,14 | ➊Figure 2 and Figure 3  ➋Additional file 2: Figure S1―S3 |
| 12 | **Assessment of assumptions** |  |  |  |
|  | a) | Report the assessment of the validity of the assumptions | 14 | ➊ Through Cochran's Q and MR-PRESSO, no significant heterogeneity (P > 0.05) or outliers were detected.  ➋ all P values of MR-Egger interpretation were > 0.05, showing the absence of horizontal pleiotropy. (Line 225―227)  ➌ no instruments were removed based on Steiger filtering (Psteiger < 0.05), and leave-one-out analysis also revealed the robustness of our main results.  ➍ above results can also be found in the Additional files |
|  | b) | Report any additional statistics (e.g., assessments of heterogeneity across genetic variants, such as I^2^, Q statistic or E-value) | 14  12 | ➊Through Cochran's Q and MR-PRESSO, no significant heterogeneity (P > 0.05) or outliers were detected.  ➋ Following the application of FDR correction, the significant result was that the genus Eubacterium fissicatena group |
| 13 | **Sensitivity analyses and additional analyses** |  |  |  |
|  | a) | Report any sensitivity analyses to assess the robustness of the main results to violations of the assumptions | 14 | ➊ Through Cochran's Q and MR-PRESSO, no significant heterogeneity (P > 0.05) or outliers were detected.  ➋ all P values of MR-Egger interpretation were > 0.05, showing the absence of horizontal pleiotropy.  ➌ no instruments were removed based on Steiger filtering (Psteiger < 0.05), and leave-one-out analysis also revealed the robustness of our main results.  ➍ above results can also be found in the Additional files |
|  | b) | Report results from other sensitivity analyses or additional analyses | 14 | no instruments were removed based on Steiger filtering (P steiger < 0.05), and leave-one-out analysis also revealed the robustness of our main results. |
|  | c) | Report any assessment of direction of causal relationship (e.g., bidirectional MR) |  | NA |
|  | d) | When relevant, report and compare with estimates from non-MR analyses |  | NA |
|  | e) | Consider additional plots to visualize results (e.g., leave-one-out analyses) |  | we performed MR visualization methods, including forest plots, leave-one-out analysis, funnel plots, and scatter plots, to evaluate the robustness of the results (Figure 3, Additional file 2: Figures. S1–S3). |
|  | **DISCUSSION** |  |  |  |
| 14 | **Key results** | Summarize key results with reference to study objectives | 15 | The present study employs GWAS datasets to conduct a two-sample MR analysis on summary statistics to genetically determine the causal relationship between the gut microbiota and PAH. Our findings indicate that the bacterial genus Eubacterium fissicatena group exhibits a causal relationship with an increased risk of PAH, whereas the genera LachnospiraceaeUCG004 and RuminococcaceaeUCG002 demonstrate a nominal causal association with PAH risk. Furthermore, we identify four additional bacterial groups, namely genus Eubacterium eligens group, genus Phascolarctobacterium, genus Erysipelatoclostridium, and genus Tyzzerella3, which exhibit nominal protective effects against PAH. |
| 15 | **Limitations** | Discuss limitations of the study, taking into account the validity of the IV assumptions, other sources of potential bias, and imprecision. Discuss both direction and magnitude of any potential bias and any efforts to address them | 18 | However, this study is subject to certain limitations, which necessitate a more cautious interpretation of the findings. The first limitation is the inability to conduct subgroup analysis, such as assessing the severity of PAH, due to the unavailability of individual level data. This is significant as alterations in intestinal flora among PAH patients were not found to be associated with changes in right heart function. In addition, the limitation of the exposure dataset to the genus level hinders our ability to investigate the causal relationship between gut microbiota and PAH at the species level. Furthermore, the predominance of participants of European descent in genome-wide association studies restricts the generalizability of our findings to other populations. Finally, while our findings establish a causal association between specific gut microbiota and PAH, further research is needed to elucidate the underlying mechanisms. |
| 16 | **Interpretation** |  |  |  |
|  | a) | Meaning: Give a cautious overall interpretation of results in the context of their limitations and in comparison with other studies |  |  |
|  | b) | Mechanism: Discuss underlying biological mechanisms that could drive a potential causal relationship between the investigated exposure and the outcome, and whether the gene-environment equivalence assumption is reasonable. Use causal language carefully, clarifying that IV estimates may provide causal effects only under certain assumptions | 16,17 | ➊The primary mechanism by which SCFAs suppress inflammation is through the inhibition of the NF-κB pathway and/or histone deacetylase (HDAC) function, resulting in the downregulation of proinflammatory cytokines such as TNF-α, IL-6, IL-12, and IFN-γ and the upregulation of anti-inflammatory cytokines such as IL-10 and TGF-β.  ➋The genus Eubacterium, as a major butyrate producer, exhibits the potential to induce anti-inflammatory effects. Eubacterium eligens is an important Eubacterium found in the human colon that promotes the production of the anti-inflammatory cytokine IL-10. In a recent study, a lower abundance of Eubacterium eligens and reductions in circulating SCFAs were observed in PAH patients. Therefore, Eubacterium eligens may produce SCFAs to exert their anti-proinflammatory effects, taken together, some gut microbiota may exert SCFA-related anti-proinflammatory effects by preventing PAH initiation and development.  ➌However, it is worth noting that not all SCFA microbial producers possess beneficial features. The families Lachnospiraceae and Ruminococcaceae possess the capacity to produce butyrate and other SCFAs through distinct biosynthetic pathways. In contrast, the findings from our MR analysis revealed that both the genus Lachnospiraceae UCG004 and the genus Ruminococcaceae UCG002 were suggestively associated with increased risks of inducing PAH. The abundance of the two taxa also exhibits an increase within the intestinal lumen of individuals afflicted with various diseases and elderly individuals. However, members of this family have consistently demonstrated their capacity to generate favorable metabolites to the host. |
|  | c) | Clinical relevance: Discuss whether the results have clinical or public policy relevance, and to what extent they inform effect sizes of possible interventions | 18 | Together, despite the presence of compelling evidence connecting gut dysbiosis to the initial development of PAH, the utilization of intestinal microbiota as a therapeutic intervention in clinical settings still requires significant advancements. It is imperative to conduct meticulous experimental investigations to establish the causative relationship between gut dysbiosis, altered gut microbiome, and the pathogenesis of PAH prior to considering the modulation of gut microbiota as a viable therapeutic approach for treating PAH. |
| 17 | **Generalizability** | Discuss the generalizability of the study results (a) to other populations, (b) across other exposure periods/timings, and (c) across other levels of exposure | 18 | Furthermore, the predominance of participants of European descent in genome-wide association studies restricts the generalizability of our findings to other populations. (Line 304―306) |
|  | **OTHER INFORMATION** |  |  |  |
| 18 | **Funding** | Describe sources of funding and the role of funders in the present study and, if applicable, sources of funding for the databases and original study or studies on which the present study is based | 20 | This work was supported by grants from the National Natural Science Foundation of China (81870049, 82170060) |
| 19 | **Data and data sharing** | Provide the data used to perform all analyses or report where and how the data can be accessed, and reference these sources in the article. Provide the statistical code needed to reproduce the results in the article, or report whether the code is publicly accessible and if so, where | 7 | The gut microbiota data utilized in this study were acquired from the international consortium MiBioGen (<http://mibiogen.gcc.nl>)  The summary statistics of PAH were downloaded from the NHGRI-EBI GWAS Catalog (<https://www.ebi.ac.uk/gwas>) on August 27, 2023 for study GCST007228. |
| 20 | **Conflicts of Interest** | All authors should declare all potential conflicts of interest | 20 | The authors declare that they have no competing interests. |

This checklist is copyrighted by the Equator Network under the Creative Commons Attribution 3.0 Unported (CC BY 3.0) license.

1. Skrivankova VW, Richmond RC, Woolf BAR, Yarmolinsky J, Davies NM, Swanson SA, et al. Strengthening the Reporting of Observational Studies in Epidemiology using Mendelian Randomization (STROBE-MR) Statement. JAMA.  2021 Oct 26;326(16):1614-1621.

2. Skrivankova VW, Richmond RC, Woolf BAR, Davies NM, Swanson SA, VanderWeele TJ, et al. Strengthening the Reporting of Observational Studies in Epidemiology using Mendelian Randomisation (STROBE-MR): Explanation and Elaboration. BMJ. 2021;375:n2233.
